# Recent advances in understanding NRF2 as a druggable target: development of pro-electrophilic and non-covalent NRF2 activators to overcome systemic side effects of electrophilic drugs like dimethyl fumarate

**DOI:** 10.12688/f1000research.12111.1

**Published:** 2017-12-14

**Authors:** Takumi Satoh, Stuart Lipton

**Affiliations:** 1 Department of Anti-Aging Food Research, School of Bioscience and Biotechnology, Tokyo University of Technology, Tokyo, Japan; 2 Neuroscience Translational Center and Department of Molecular Medicine, The Scripps Research Institute, La Jolla, CA, USA; 3 Neurodegenerative Disease Center, Scintillon Institute, San Diego, CA, USA; 4 Department of Neurosciences, University of California, School of Medicine, La Jolla, CA, USA

**Keywords:** Nrf2, Dimethyl Fumarate, Electrophilic Drugs, KEAP1

## Abstract

Dimethyl fumarate (DMF) is an electrophilic compound previously called BG-12 and marketed under the name Tecfidera ^®^. It was approved in 2013 by the US Food and Drug Administration and the European Medicines Agency for the treatment of relapsing multiple sclerosis. One mechanism of action of DMF is stimulation of the nuclear factor erythroid 2-related factor 2 (NRF2) transcriptional pathway that induces anti-oxidant and anti-inflammatory phase II enzymes to prevent chronic neurodegeneration. However, electrophiles such as DMF also produce severe systemic side effects, in part due to non-specific S-alkylation of cysteine thiols and resulting depletion of glutathione. This mini-review presents the present status and future strategy for NRF2 activators designed to avoid these side effects. Two modes of chemical reaction leading to NRF2 activation are considered here. The first mode is S-alkylation (covalent reaction) of thiols in Kelch-like ECH-associated protein 1 (KEAP1), which interacts with NRF2. The second mechanism involves non-covalent pharmacological inhibition of protein-protein interactions, in particular domain-specific interaction between NRF2 and KEAP1 or other repressor proteins involved in this transcriptional pathway. There have been significant advances in drug development using both of these mechanisms that can potentially avoid the systemic side effects of electrophilic compounds. In the first case concerning covalent reaction with KEAP1, monomethyl fumarate and monoethyl fumarate appear to represent safer derivatives of DMF. In a second approach, pro-electrophilic drugs, such as carnosic acid from the herb *Rosmarinus officinalis*, can be used as a safe pro-drug of an electrophilic compound. Concerning non-covalent activation of NRF2, drugs are being developed that interfere with the direct interaction of KEAP1-NRF2 or inhibit BTB domain and CNC homolog 1 (BACH1), which is a transcriptional repressor of the promoter where NRF2 binds.

## The KEAP1/NRF2 pathway

Neurons are highly sensitive to the balance system between oxidation and reduction, and the disruption of this system can lead to inflammatory reactions contributing to various acute and chronic diseases as well as to the normal aging process ^1, 2^. Activation of the KEAP1/NRF2/anti-oxidant-response element (ARE) pathway by electrophiles (EPs) can activate this cellular redox defense system against these diseases ^2, 3^. The NRF2/KEAP1 pathway represents one of the major cellular defense systems against oxidative stress, inflammatory reactions, and exposure to toxic electrophilic compounds ^4– 7^. NRF2 is a transcription factor that induces various anti-oxidant, anti-inflammatory, and detoxification enzymes ^4– 7^. Under physiological conditions, KEAP1 protein binds to NRF2 and functions as an adaptor protein for cullin 3 (encoded by *Cul3* in humans) E3 ubiquitin ligase, which polyubiquitinates NRF2. Consequently, NRF2 is ubiquitinated and degraded by the proteasome ^4– 7^. Hence, the transcriptional activity of NRF2 is potently inhibited under normal conditions ^4– 7^.

KEAP1 contains critical cysteine thiols that react with endogenous and exogenous EPs ^6, 8– 11^. This reaction reduces the ability of KEAP1 to induce ubiquitination and degradation of NRF2 ^6, 8– 11^. After EP reaction, NRF2 dissociates from the cytoplasmic complex with KEAP1, enters the nucleus, and accumulates there to drive transcription of its target phase II genes, which encode a coordinated system of anti-oxidant and anti-inflammatory enzymes. These proteins include enzymes that generate the major cellular anti-oxidant, glutathione (GSH) ^6, 8– 11^. Thus, NRF2 activators have been shown to be anti-inflammatory and neuroprotective at least in part via redox regulation ^6, 8– 11^.

Additionally, NRF2 activators can potently induce coordinated expression of genes involved in the autophagy system, including p62 ^12– 14^. In turn, p62 protein then activates the NRF2/ARE pathway, representing a positive feedback loop between the NRF2/ARE pathway and autophagy network ^12– 14^. By simulating autophagy in this fashion, NRF2 activators can potentially remove misfolded proteins and thus suppress several diseases associated with abnormal protein conformation ^12– 14^. NRF2 activators have also been suggested to be neuroprotective against Alzheimer’s disease (AD), Parkinson’s disease (PD), and Huntington’s disease (HD) ^6, 8– 14^ on the basis of results in animal models of these neurodegenerative disorders.

During oxidative stress, p62 expression is enhanced via an NRF2-mediated mechanism. The increased p62 can interfere with NRF2/KEAP1 binding and thus results in a positive feedback loop, increasing NRF2 activation ^12– 14^. The detailed mechanism of p62-KEAP1-NRF2 interaction remains contentious, but some possible scenarios have been proposed ^13^. For example, p62 has a STGE motif in its KEAP1-interacting domain and thus p62 may directly bind to KEAP1. The p62 STGE motif may potentially compete with the NRF2 ETGE motif, which is essential for KEAP1-NRF2 interaction ^13^. When p62 is upregulated by NRF2 under oxidative stress, p62 then may compete out NRF2 from the KEAP1-NRF2 complex, thus allowing NRF2 to translocate into the nucleus and activate the ARE in the promoter region of phase II genes ^13^.

However, some NRF2 activators that upregulate p62, such as arsenic, may result in impairment of autophagy, and p62 activation of NRF2 often occurs in the setting of autophagy impairment ^13^. Thus, increased p62 can be associated with impairment of autophagy rather than facilitation ^12^. Although NRF2 controls the expression of several autophagy-related genes ^14^, the functional linkage between NRF2 and these putative target autophagy genes under physiological or pathophysiological conditions remains to be determined.

NRF2 manifests both positive and negative attributes with respect to cancer and other diseases ^15, 16^. On the one hand, NRF2 activators have been proposed for the treatment of various forms of cancer ^6, 8, 9^. In contrast, other recent investigations based on genetic findings suggest that NRF2 activation can promote neoplasia, possibly by enhancing resistance to cancer treatment ^15, 16^. For example, gain-of-function mutations in NRF2 and loss-of-function mutations in KEAP1 have been encountered in tumors of the digestive tract ^15, 16^. Further investigation is merited to clarify the biological significance of NRF2 activation in cancer ^15, 16^.

## Cysteine-mediated regulation of KEAP1

Among the cysteine thiols of KEAP1 protein, the most characterized reactive thiols are Cys151, Cys273, and Cys288, and they have differential roles in the activation of the KEAP1/NRF2 pathway. The major cysteine residues of KEAP1 that react with EPs are Cys151, Cys273, and Cys288. Each of these cysteine thiols may differentially regulate phase II anti-oxidant gene expression stimulated by the KEAP1/NRF2 transcriptional pathway ^17, 18^.

For example, KEAP1 Cys151 contains the most important thiol for activation of the KEAP1/NRF2 transcriptional pathway ^18, 19^. Located in the N-terminal BTB domain, Cys151 may be very reactive because of a stretch of basic amino acids in the α5 helical structure ^19, 20^. One model suggests that covalent modification of Cys151 causes dissociation of the KEAP1/Cullin3 heterodimer, resulting in inhibition of NRF2 ubiquitination ^19, 20^. Reaction of Cys151 with EPs is thus critical for inhibition of NRF2 degradation mediated by KEAP1-dependent degradation of NRF2 ^19– 21^. In contrast, mutation of KEAP1 Cys151 produces constitutive inhibition of NRF2 under both physiological and pathological conditions in cell-based assays ^22, 23^. Additionally, ubiquitination and degradation of NRF2 require cysteine residues 273 and 288 of KEAP1. Previous studies of mutations revealed that substitution of Cys273 or Cys288 prevented KEAP1 from repressing NRF2 activity under homeostatic conditions ^24– 26^.

## DMF/MMF/MEF

### Dimethyl fumarate

Dimethyl fumarate (DMF) is currently approved for clinical use by the US Food and Drug Administration (FDA) and the European Medicines Agency for the treatment of relapsing multiple sclerosis (MS) ^27, 28^. DMF is an alkylating agent, similar to the classic NRF2 activator sulforaphane, which can non-specifically and covalently modify nucleophilic groups in proteins, including cysteine thiols ^29, 30^. As a result, serious side effects can occur with this type of drug. For example, a 30% decline in lymphocyte counts has been reported after administration of DMF, which may predispose to infection ^31– 34^. DMF has two congeners: monomethyl fumarate (MMF) and monoethyl fumarate (MEF). Recent research interest has shifted to MEF and MMF with the hope of developing a safer drug than DMF because both of these congeners are less electrophilic than DMF ^35– 38^. DMF has also been shown to react with other thiol targets, which appear to predominate over KEAP1, at least in T cells ^39^.

### Monoethyl fumarate

**Figure 1.**
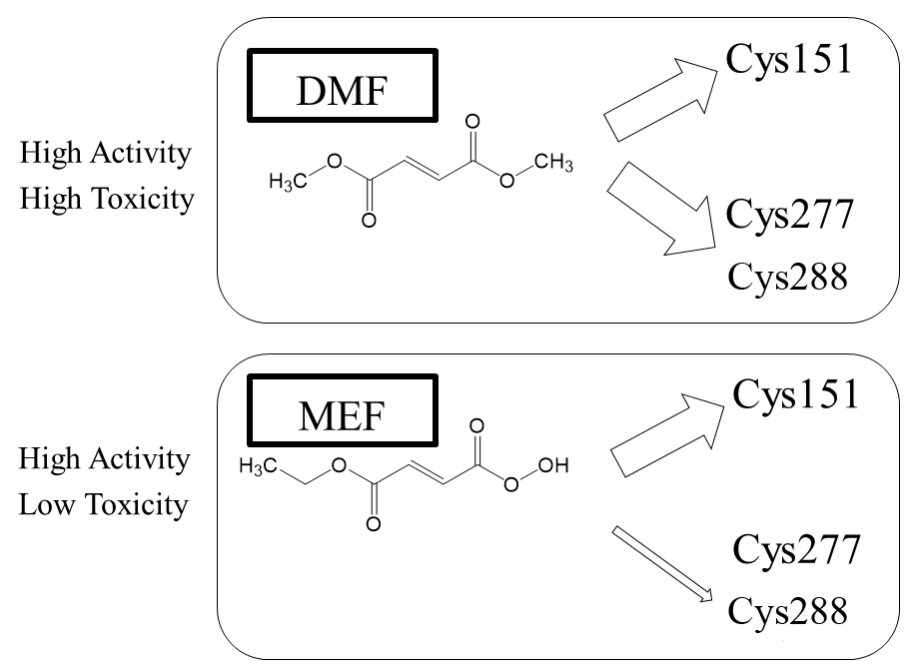
DMF and MEF modulate distinctive repertoires of cysteine thiols on KEAP1. Although DMF reacts with many cysteine residues, including Cys151, Cys273, and Cys288, MEF appears to react preferentially with Cys151. DMF has proven to be more toxic than MEF, although DMF and MEF both activate NRF2, at least *in vitro* ^35, 36^. DMF, dimethyl fumarate; KEAP1, Kelch-like ECH-associated protein 1; MEF, monoethylfumarate; NRF2, nuclear factor erythroid 2-related factor 2.

DMF and MEF react with disparate KEAP1 thiols, and DMF is more reactive toward a larger number of cysteines ^35– 39^. MEF appears to solely modulate Cys151 on KEAP1 and manifests significantly less reaction with other KEAP1 cysteines compared with DMF ( Figure 1) ^35, 36^. On the other hand, DMF induces greater NRF2 protein accumulation than MEF ^35, 36^. Potentially accounting for some of its side effects, DMF has also been shown to acutely deplete GSH in a concentration-dependent manner ^32, 34, 35, 39^. In contrast, MEF maintains GSH levels and, in fact, may produce an increase, possibly due to NRF2 stimulation of GSH synthetic enzymes ^35, 36^. Thus, MEF may prove to be less toxic than DMF ^35, 36^.

### Monomethyl fumarate

**Figure 2.**
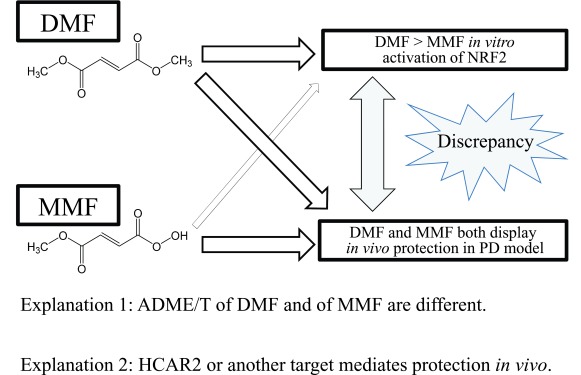
Discrepancy between *in vivo* and *in vitro* actions of DMF and MMF in murine PD models. DMF and MMF show comparable protective action in an *in vivo* rodent model of PD. In contrast, MMF is far less potent than DMF in terms of *in vitro* NRF2 activation ^37, 38^. There are at least two possible explanations for this discrepancy. One possible interpretation is that DMF and MMF display differential ADME/T (absorption, distribution, metabolism, excretion, toxicity) parameters *in vivo* ^37, 38^. Another possible explanation is that reaction with HCAR2 or another target mediates the protective effects by DMF ^37, 38^. DMF, dimethyl fumarate; HCAR2, hydroxycarboxylic acid receptor 2; MMF, monomethylfumarate; NRF2, nuclear factor erythroid 2-related factor 2; PD, Parkinson’s disease.

A recent study demonstrates similar therapeutic benefits for DMF and its bioactive metabolite MMF in a rat model of PD and brain stroke ^37, 38^. Despite their similar pharmacological effects *in vivo*, MMF is a less potent NRF2 activator and manifests less toxicity *in vitro*, probably because it manifests orders of magnitude less non-specific alkylating capacity than DMF ( Figure 2) ^37, 38^. The discovery of the therapeutic effects of MMF in an experimental PD model without substantial non-specific alkylating properties compared with DMF suggests that MMF may be a candidate for PD and stroke therapeutics ^37, 38^. MEF may also potentially be considered as a therapeutic agent since its alkylating capacity is also low like that of MMF ^35– 38^. Nonetheless, the lack of specificity of these alkylating NRF2 activators with regard to other protein thiol targets as well as further consideration of their pharmacokinetic and pharmacodynamic properties may limit their ultimate usefulness ^37, 38^.

### Hydroxycarboxylic acid receptor 2 as an alternate target

Other experiments suggest that HCAR2 activation, rather than NRF2 activation, may be partially responsible for the beneficial action of DMF and MMF in PD and MS models ^40, 41^. HCAR2 is a G protein–coupled receptor whose ligands are hydroxyl-carboxylic acids produced from energy metabolism in order to sense cellular metabolic status ^40, 41^. HCAR2 is expressed in a number of immune cells and other cell types ^40, 41^. Emerging evidence suggests that HCAR2 exerts potentially therapeutic anti-inflammatory actions ^40, 41^. Along these lines, in *Hcar2* ^-/-^ mice, the beneficial effect of DMF in a mouse model of MS (autoimmune encephalomyelitis or experimental autoimmune encephalomyelitis) is completely abrogated, consistent with the notion that HCAR2 plays an important role in the effect of DMF ^40, 41^. Anti-inflammatory effects of DMF in the brain have also been posited to be NRF2-dependent, at least in part ^42^. If HCAR2 is indeed a major therapeutic target of DMF in AD, PD, and HD, then the ketone body ß-hydroxybutyrate, a known HCAR2 ligand, may prove to be a more suitable therapeutic than DMF, MEF, or MMF ^43, 44^. Hence, additional thiol targets of DMF and related compounds are a major focus of current studies.

## Pro-electrophilic drugs as pathologically activated therapeutic drugs

### Pro-electrophilic drugs

**Figure 3.**
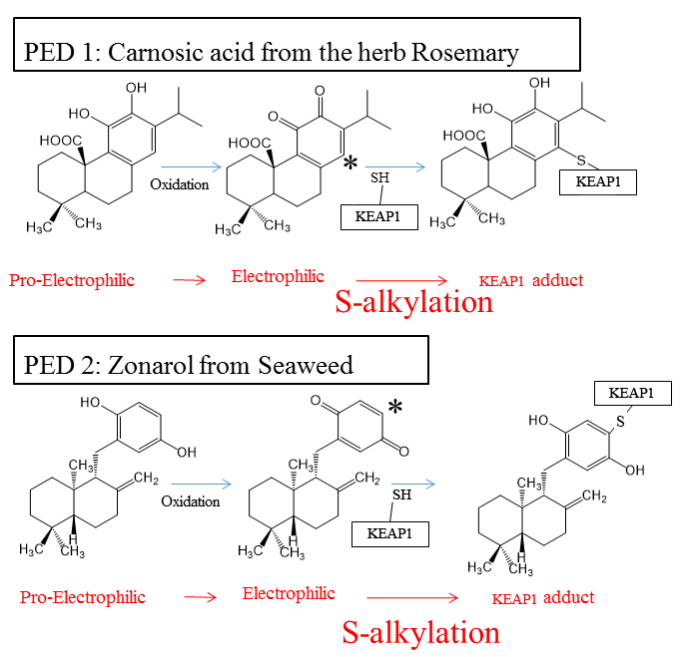
Activation of the KEAP1/NRF2 pathway by PEDs (PED 1, CA; PED 2, ZO). The PED compounds CA (with adjacent or “ *ortho*-” position hydroxyl groups) ^46, 47, 49, 50^ and ZO (with hydroxyl groups located directly across the ring, in the “ *para*-” position) ^51, 52^ become oxidized to the electrophilic *quinone* form. CA and ZO quinones undergo nucleophilic attack by a critical KEAP1 cysteine thiol. The reaction forms a KEAP1-CA or KEAP1-ZO adduct. This results in release of NRF2 from KEAP1/NRF2 complexes, accumulation of NRF2 in the nucleus, and subsequent transcriptional activation of phase II enzymes ^45, 46^. Phase II anti-oxidant and anti-inflammatory enzymes reduce reactive oxygen species and thus improve the resilience of neurons. Importantly, the oxidation of hydroquinone (PED) to quinone (EP) is triggered by oxidative stress, which is then combatted by this transcriptional activity, as described in the text ^45, 49^. CA, carnosic acid; EP, electrophile; KEAP1, Kelch-like ECH-associated protein 1; NRF2, nuclear factor erythroid 2-related factor 2; PD, Parkinson’s disease; PED, pro-electrophilic drug; ZO, zonarol.

Redox imbalance (for example, excessive oxidation over reduction) is believed to contribute to a variety of diseases ^1^. Prior use of EPs to improve redox balance by activating transcriptional systems against oxidative stress has been met with mixed success, largely because of side effects due to the indiscriminate action of EPs ^2^. A newer approach uses pro-drug forms of EPs, known as pro-electrophilic drugs (PEDs), such as carnosic acid (CA), an active ingredient in the herb rosemary ( *Rosmarinus officinalis*) ^45– 50^. Additional compounds of interest include zonarol (ZO) and isozonarol (IZ), which are found in seaweed ( *Dictyopteris undulata*) ( Figure 3) ^51, 52^, as well as related synthetic chemicals ^53, 54^. Importantly, these PEDs do not react directly with cysteine thiols. However, oxidative stress triggers their conversion from hydroquinone to quinone, representing an active EP. The EP then triggers KEAP1/NRF2/ARE transcriptional activity, resulting in the production of anti-oxidant/anti-inflammatory phase II enzymes ^45, 49^.

The combined efforts of the authors’ research groups have led to the development of PEDs that are activated by the very oxidative stress that they then serve to counteract. This type of action has been deemed a pathologically activated therapeutic or ‘PAT’ drug ^55, 56^—a drug that is active only at the site where it is needed and thus represents a gentle tap or pat compared with more indiscriminant reagents that are reactive throughout the body, such as more conventional EPs ^45, 49^. Since PEDs are not activated in normal cells, they do not indiscriminately react with other thiols such as GSH; moreover, the cells undergoing oxidative stress in which PEDs are converted to EPs already display depleted levels of GSH; hence, the EP generated from the PED does not encounter the normally high levels of GSH with which to react ^45, 49^. This type of action may help to minimize the side effects of PEDs while retaining beneficial activity ^48, 49^. Thus, the anti-oxidant NRF2-activating therapy of PEDs is targeted only to cells ‘in need’. Additionally, owing to their stimulation of a transcriptional pathway producing endogenous anti-oxidant enzymes, PEDs exhibit a more sustained and amplified action than standard anti-oxidant compounds ^45, 48^. Accordingly, our recent neurobehavioral and histological readouts suggest that CA, acting as a PED, and administered orally, transnasally, or parenterally *in vivo*, can be an effective treatment for AD and other neurologic conditions in rodent models ^46, 47, 50^.

## Inhibitors of protein-protein interaction

### KEAP1-NRF2 PPI

NRF2 has a Neh2 domain in its N-terminal regulatory region, which is important for binding to the Kelch-DC domain of the C-terminus of KEAP1 ^17– 20^. Peptides capable of blocking the KEAP1-NRF2 protein-protein interaction (PPI) have been identified and proven to be protective in models of global ischemia ^57, 58^. Importantly, this non-covalent mechanism of action is completely different from electrophilic NRF2 activators, which react at Cys151 of the N-terminal domain of KEAP1 in a covalent manner ^17– 20^. Recent structural and functional studies have further illuminated the details of the non-canonical mechanism of NRF2 activation ^17– 20^. The Kelch-DC domain of KEAP1 binds to NRF2 via either its DLG or ETGE motif; both of these motifs are thought to be the major targets of non-covalent inhibitors of KEAP1-NRF2 PPI ^59, 60^. In a hinge-and-latch model of this interaction, the ETGE motif has a higher affinity for KEAP1 than the DLG motif, which causes the latter to associate and dissociate from KEAP1 in a dynamic manner, resulting in oscillations between a ‘closed’ (associated) and ‘open’ (dissociated) conformation ^59, 60^.

### KEAP1-NRF2 PPI inhibitors

**Figure 4.**
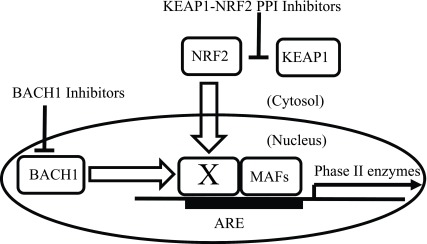
Two types of PPI inhibitors can activate NRF2. NRF2-KEAP1 PPI inhibitors directly inhibit binding of NRF2 and KEAP1 proteins and result in NRF2 release, translocation into the nucleus, and activation of phase II gene transcription ^59, 60^. Under physiological conditions, BACH1 constitutively inhibits NRF2-mediated transcriptional activity ^68– 71^. BACH1 inhibitors bind to BACH1 ^68– 71^. Thus, BACH1 inhibitors can activate transcription of NRF2-dependent phase II genes ^74^. In this figure, the “X” designates a partner of sMAFs ^72, 73^. X and sMAFs can form homo- or hetero-dimers and bind to ARE elements ^72, 73^. When “X” is a sMAF or BACH1, phase II enzymes are not induced; in contrast, when “X” is NRF2, phase II enzymes are induced ^72, 73^. ARE, anti-oxidant-response element; BACH1, BTB and CNC homology 1; KEAP1, Kelch-like ECH-associated protein 1; NRF2, nuclear factor erythroid 2-related factor 2; PPI, protein-protein interaction; sMAF, small musculoaponeurotic fibrosarcoma protein.

Non-electrophilic NRF2 activators have been proposed as therapeutic agents for chronic neurodegeneration and inflammation because of their potentially lower incidence of side effects compared with EPs ( Figure 4) ^59, 60^. Using peptide displacement for high-throughput screening, small molecules have been identified that interfere with KEAP1-NRF2 binding ^57– 60^. Accordingly, KEAP1-NRF2 PPI inhibitors are being studied as NRF2 activators in several disease models ^6, 61, 62^. Taking advantage of this molecular mechanism of action should allow chemists to optimize such agents for the development of non-covalent NRF2 activators ^63– 67^. To date, many studies of KEAP1-NRF2 PPI inhibitors have focused on the KEAP1-NRF2 ETGE motif ^59, 60^. However, the affinity of this binding reaction is very high and difficult to inhibit ^59, 60^. In contrast, as alluded to above, the KEAP1-NRF2 DLG interaction is weaker and has rapid association and dissociation rates ^59, 60^. Thus, inhibition of binding at the KEAP1-NRF2 DLG may represent an improved approach to further develop effective KEAP1-NRF2 PPI inhibitors ^59, 60^. Another possible target is the p62 STGE motif, which can compete with the NRF2 ETGE motif for binding to KEAP1 ^12– 14^.

### BTB and CNC homology 1 inhibitors

Yet another mechanism for ARE-mediated gene regulation involves BACH1, which functions as an inhibitor of NRF2-mediated transcription by binding to small musculoaponeurotic fibrosarcoma proteins (sMAFs) and occupying ARE promoter elements ^68– 71^. As shown in Figure 4, the basic concept of BACH1 inhibition is competition between BACH1 and NRF2 for dimer formation with sMAFs on ARE-containing promoters ^68– 71^. In essence, BACH1 inhibitors serve to inhibit the action of an inhibitor, resulting in NRF2 activation. sMAFs are leucine zipper–type transcription factors containing basic regions ^72, 73^. The basic region of sMAF family members contributes to the distinct DNA-binding mode of this class of proteins ^72, 73^. sMAFs form homodimers as well as heterodimers with NRF2 or BACH1 ^72, 73^. Because NRF2 and BACH1 cannot bind to DNA as monomers, sMAFs are indispensable partners in order to bind to ARE-containing promotors. In contrast, sMAF homodimers basically act as transcriptional repressors ^72, 73^. Additionally, binding of heme to BACH1 will displace this repressor, allowing it to be degraded ^68– 71^. As expected, BACH1 gene knockout results in activation of the KEAP1/NRF2 pathway and protection in various disease models ^68– 71^. Hence, the development of drugs that bind BACH1 could also contribute to activation of NRF2-dependent phase II enzymes and prove therapeutic in the future ^70, 74^.

## Summary

In conclusion, new forms of both covalent and non-covalent NRF2 activators have recently shown promise as protectants from neurologic diseases; they may also be beneficial for other cell types affected in systemic diseases, including type 2 diabetes mellitus and possibly even normal aging. The new compounds offer hope of efficacy without indiscriminately reacting with protein thiols, which contribute to the multiple side effects observed with the older EP-like drugs, including curcumin and DMF. Recently, excitement has been generated over the possibility of developing non-covalent NRF2 activators. However, the pathologically targeted covalent-reacting PED, CA, appears on the ‘generally regarded as safe’ (GRAS) list approved by the FDA and has been consumed in large quantities by humans for over two thousand years without incident. It is not yet clear whether the newer non-covalent NRF2 activators will be as well tolerated by humans and avoid systemic toxicity. Considerable further discovery, optimization, and clinical testing will be needed to bring these new drugs to market for neurological as well as systemic diseases.

## Abbreviations

AD, Alzheimer’s disease; ARE, anti-oxidant-response element; BACH1, BTB and CNC homology 1; CA, carnosic acid; DMF, dimethyl fumarate; EP, electrophile; FDA, US Food and Drug Administration; GSH, glutathione; HCAR2, hydroxycarboxylic acid receptor 2; HD, Huntington’s disease; KEAP1, Kelch-like ECH-associated protein 1; MEF, monoethylfumarate; MMF, monomethylfumarate; MS, multiple sclerosis; NRF2, nuclear factor erythroid 2-related factor 2; PD, Parkinson’s disease; PED, pro-electrophilic drug; PPI, protein-protein interaction; sMAF, small musculoaponeurotic fibrosarcoma protein.
